# Renal Failure Prevalence in Poisoned Patients

**DOI:** 10.5812/numonthly.11910

**Published:** 2014-03-11

**Authors:** Mohammad Arefi, Fakhroddin Taghaddosinejad, Peyman Salamaty, Davood Soroosh, Hami Ashraf, Mohsen Ebrahimi

**Affiliations:** 1 Department of Forensic Medicine, Tehran University of Medical Sciences, Tehran, IR Iran; 2 Sina Trauma and Surgery Research Center, Tehran University of Medical Sciences, Tehran , IR Iran; 3 Department of Forensic Medicine, Mashhad University of Medical Sciences, Mashhad, IR Iran; 4 Razavi Hospital, Mashhad, IR Iran; 5 Department of Emergency Medicine, Mashhad University of Medical Sciences, Mashhad, IR Iran

**Keywords:** Drug-Related Side Effects and Adverse Reactions, Renal Insufficiency, Rhabdomyolysis

## Abstract

**Background:**

Renal failure is an important adverse effect of drug poisoning. Determining the prevalence and etiology of this serious side effect could help us find appropriate strategies for the prevention of renal failure in most affected patients.

**Objectives:**

The present study is aimed to identify drugs that induce renal failure and also to find the prevalence of renal failure in patients referred to emergency departments with the chief complaint of drug poisoning, in order to plan better therapeutic strategies to minimize the mortality associated with drug poisoning induced renal failure.

**Patients and Methods:**

This cross-sectional study surveyed 1500 poisoned patients referred to the Emergency Department of Baharloo Hospital in Tehran during 2010. Demographic data including age and gender as well as clinical data including type of medication, duration of hospital stay, and presence of renal failure were recorded. Mann-Whitney U test and chi-squared statistics were used to analyze the results.

**Results:**

A total number of 435 patients were poisoned with several drugs, 118 patients were intoxicated with sedative-hypnotic drugs, 279 patients were exposed to opium, and 478 patients were administered to other drugs. The method of intoxication included oral 84.3%, injective 9%, inhalation 4.3% and finally a combination of methods 2.3%. Laboratory results revealed that 134 cases had renal failure and 242 had rhabdomyolysis. The incidence of rhabdomyolysis and renal failure increased significantly with age, and also with time of admission to the hospital. Renal failure was reported in 25.1% of patients exposed to opium, vs. 18.2% of patients poisoned with aluminum phosphide, 16.7% of those with organophosphate, 8% with multiple drugs, 6.7% with alcohol, heavy metals and acids, and 1.7% with sedative hypnotics.

**Conclusions:**

Based on the findings of this study, there is a high probability of renal failure for patients poisoned with drugs such as opium, aluminum phosphide, and multiple drugs as well as the patients with delayed admission to the hospital, and it is necessary to seek appropriate treatment to prevent this significant side effect.

## 1. Background

Since a significant percentage of poisoned patients are referred to the emergency departments of hospitals (15-20%), immediate attention to drug toxicity and its clinical signs and symptoms is required at the first step. Previous studies have shown that drug and chemical poisoning is a common and serious clinical problem worldwide. In the United States of America, accidental poisoning with the chemicals leads to an annual 5000 mortality rate (1). Acute renal failure is determined by a sharp decline in glomerular filtration rate (GFR) within a few hours to a full day. Depending on the precise definition of renal failure nearly 5% to 7% of hospitalized cases and 30% of patients admitted to hospital intensive care unit are affected by it. Acute renal failure can be a complication of exposure to many pharmacological and substances, such as X-ray contrast agents, cyclosporine, tacrolimus, anti-cancer drugs, antibiotics and aminoglycoside antibiotics, opium, and organophosphates (2).

If reliable criteria to determine the risk factors are available, an early prognosis of the patient can be assumed. Knowing drugs associated with high risks of causing renal failure, reduction of renal failure and its associated mortality rates will be possible. Derakhshan and Moadab in a prospective study reported that gentamicin, amikacin, and nephrotoxic drugs like vancomycin are the most common causes of acute tubular necrosis in pediatric poisoning and children on these drugs must receive excessive attention (3). In a recent study conducted by Toth and Dayspring, 120 patients diagnosed with any type of renal disease were treated with rosuvastatin (4). In another study by Hall et al. it was concluded that if a patient ingested less than 6 grams of ibuprofen, renal testing is not required (5). Also Roy et al. reported that in 2500 patients who were chronic users of lithium, 20% had diabetes insipidus (6). Similarly Shahbazian reported that vitamin D can lead to acute renal failure (7).

## 2. Objectives

The present study is aimed to identify drugs inducing renal failure and also to find the prevalence of renal failure in patients referred to emergency departments with the chief complaint of drug poisoning in order to plan better therapeutic strategies to minimize the mortality associated with drug poisoning induced renal failure.

## 3. Patients and Methods

A total number of 1500 patients, referred to emergency epartment of Baharloo Hospital, Tehran University of Medical Sciences, during year 2010 were enrolled to this descriptive cross-sectional study. Renal function test results were evaluated for all poisoned patients. A detailed medical history was obtained from the patients or their companion relatives and physical examination was performed by a medical doctor. The laboratory tests including urine or serum drug screen were studied as well. Laboratory tests for rhabdomyolysis including creatine phosphokinase (CPK) and lactate dehydrogenase (LDH) were also measured. Necessary information such as the type of drug, drug dosage, and time elapsed from drug indigestion to admission of the patient, the treatment process, and presence or lack of renal failure and rhabdomyolysis were registered. Patients with previous definite renal failure and the ones not having all laboratory results required to detect renal failure were excluded from further analyzes.

In this study patients exposed to drug toxicity were evaluated. Renal failure was defined as having a creatinine (Cr) level greater than 1.4 mg/dL and rhabdomyolysis defined as CPK and LDH results at least five times higher than normal levels for the same gender and age (8, 9). Patients who showed strong evidence of renal failure were excluded from the study. This study is approved by the research deputyship of Tehran University of Medical Sciences regarding ethical and methodological issues. A written consent was obtained from each individual or their parents after giving full description of the aims of the study. The data were registered and analyzed using SPSS version 18.0. Mann-Whitney U test and Chi-squared statistics were employed to analyze the results. Related tables were produced to discuss the results. P values ≤ 0.05 were considered as statistically significant.

## 4. Results

Among 1500 patients, 780 patients (52%) were male and 720 (48%) were female. The mean age of patients was 30.55 years, with a standard deviation of 11.955 (minimum 14 and maximum 69 years old). Among all patients, 435 cases were poisoned with multiple drugs (29%), 308 cases (20.5%) with Sedative Hypnotic Drugs, 279 cases with opium (18.6%) 60 cases (4%) with alcohol and heavy metals, 55 cases with phosphide (3.7%), 53 cases with tricyclic antidepressants (TCA) (3.5%), and 50 cases with acetaminophen (3.3%). One thousand and two hundred sixty five patients (84.3%) used the drug orally, 135 (9%) intravenously, 65 (3.4%) consumed by inhalation, and 35 (3.2%) combined oral and intravenous use. One hundred and forty five patients (9.7%) were admitted to the hospital within one hour, 860 patients (57.3%) between 1-5 hours after ingestion, 475 patients (31.7%) between 5-24 hours after consumption, and 20 patients (1.3%) after 24 hours. From the total 1500 patient 134 patients (8.9%) had renal failure with creatinine greater than 1.4 mg/dL. Also 242 (16.1%) patients had rhabdomyolysis with over five folded CPK and LDH levels than the normal values.

Mann-Whitney U test showed a significant correlation between age and renal failure, implying that the prevalence of renal failure increases with age (Table 1). Moreover, there is a statistically significant relationship between gender and renal failure. A greater proportion of males were diagnosed with renal failure (Table 2). Among 17 groups of medications, multiple drugs (29.0%, n = 435 patients) had the highest frequency followed by sedative hypnotic drugs (20.5%, n = 308 patients), and opium (18.6%, n = 279 patients). The drugs that had the highest chance of causing renal failure were opium (25.1%), aluminum phosphide (18.2%), organophosphates (16.7%), multiple drugs (8%), alcohol, heavy metals and acids (8%), and sedative hypnotics (1.7%) respectively. Among 242 patients with rhabdomyolysis, 69 (28.5%) patients experienced renal failure. Among 1258 patients without rhabdomyolysis, 65 (5.2%) patients experienced renal failure. However, there is a definite relationship between rhabdomyolysis and renal failure. The patients with rhabdo myolysis generally had a higher risk of developing renal failure (Table 2). There was also a significant correlation between time interval between drug poisoning and arrival to the hospital and the presence of renal failure and rhabdomyolysis. The later the patient was admitted to the hospital, the higher the chance of renal failure and rhabdomyolysis were (P value < 0.001) (Tables 2 and 3).

**Table 1.** Relation Between Renal Failure and Rhabdomyolysis and Age in Patients of This Study ^a^

	Results	Age, y	P value ^b^
**Renal failure**			< 0.001
Present	134 (8.9)	38.75 ± 13.399	
Absent	1366 (91.1)	29.75 ± 11.499	
**Rhabdomyolysis**			< 0.001
Present	242 (16.1)	33.75 ± 12.619	
Absent	1258 (83.9)	30.03 ± 11.757	

^a^ Data are presented in No. (%) or Mean ± SD.

^b^ Mann-Whitney test.

**Table 2.** Relation Between Sex, Renal Failure, Rhabdomyolysis, and Time Elapsed From Drug Ingestion to Hospital Admittance ^a^

	Renal Failure		P value
	Yes	No	
**Gender**			
Male	100 (12.8)	680 (87.2)	< 0.001
Female	34 (4.7)	686 (95.3)	
**Rhabdomyolysis**			
Yes	69 (28.5)	173 (71.5)	< 0.001
No	65 (5.2)	1193 (94.8)	
**Time between drug indigestion and refer, h**			
< 1	10 (6.9)	135 (93.1)	< 0.001
1-5	50 (5.8)	810 (94.2)	
5-24	65 (13.7)	410 (88.3)	
> 24	9 (45.0)	11 (55.0)	
**Treatment**			
On time	45 (4.6)	925 (95.4)	< 0.001
Not on time	89 (16.8)	441 (83.2)	

^a^ Data are presented in No. (%).

**Table 3.** Evaluation of Rhabdomyolysis in Relation to Time Between Ingestion and Receiving or Not ^a^

	Rhabdomyolysis		P value
	Yes	No	
**Time from use to hospital admittance, h**			< 0.001
< 1	15 (10.3)	130 (89.7)	
1-5	89 (10.3)	771 (89.7)	
5-24	128 (26.9)	347 (73.1)	
> 24	10 (50)	10 (50)	
**Proper treatment**			< 0.001
On time	50 (5.2)	920 (94.8)	
Not on time	192 (36.2)	338 (63.8)	

^a^ Data are preseted in No. (%).

Most patients (860 patient, 57%) were hospitalized between 1-5 hours after drug consumption, while 7.9% (n = 145) were hospitalized in less than one hour, 31% (n = 475) between 5-24 hours, and only 3.1% (n = 20) were admitted after 24 hours. Besides, 7.64% of the patients were treated with gastrointestinal decontamination along with the use of activated charcoal, and appropriate antidotes and 3.35% did not receive these treatments on time.

## 5. Discussion

This study showed that number of male patients poisoned was slightly greater than women (780 males vs. 720 females). Higher prevalence of addiction and suicide in men may be directly correlated to drug abuse. This may indicate that men may need more preventive education. Shadnia et al. in Loqman Hospital reported that among 10206 hospitalized poisoned patients, 51% were male and 49% were female (10). In another study in 2009 in Shanghai, among 374 patients with drug induced renal failure 65% were male and 35% were female (11). The mean age of patients in this study was 30.55 years with a standard deviation of 11.95. In a study conducted in London 38% of patients were 21 to 30 years old (10) while In Shanghai 51% were over 60 years old (11). Multiple drugs poisoning, sedative hypnotic drugs, and opium, form the majority of abused substances. Therefore improving medical personnel knowledge about the different aspects of these drugs with proper education seems imperative.

In the present study 860 patients (57%) were hospitalized between 1-5 hours after drug consumption, while only 9.7% (n = 145) were hospitalized in less than one hour. Similar studies reported the time interval between the accidental drug consumption and admission to hospital as less than one hour (12). The prevalence of drug-induced renal failure in patients of the study was 8.9% (n = 134) which is relatively high comparing to similar studies. In a study conducted in the United States of America 15.6% of the patients showed drug induced renal failure (13). Also In another study in India, for 10% of patients, anti-TB drugs poisoning led to renal failure (14). Therefore it is important during treatment of drug toxicity that medical personnel pay particular attention to the probability of developing renal failure and to follow up with proper treatment. In this study, the incidence of rhabdomyolysis was 16.1 % (242 people) which is relatively high and an urgent need for an effective prevention strategy is felt. Only in one study in 2006, incidence of rhabdomyolysis in olanzapine poisoning was reported as 17% (15). The present study showed that renal failure incidence increases with age (P < 0.001) indicating that the age is an important risk factor for renal failure. Older individuals need more attention, especially in serum therapy in conditions with volume depletion. In a study conducted in 2011 in Brussels age over 60 years was determined to be the risk factor for renal failure (16). The incidence of renal failure in patients with rhabdomyolysis was 28.5% while 5.2% in patients without it which shows a high risk of renal failure in patients with rhabdomyolysis (P < 0.001). Therefor rhabdomyolysis is a strong risk factor for renal failure and the prompt and proper treatment to prevent rhabdomyolysis is important in further prevention of renal failure. Type of medications causing drug induced renal failure was also determined and opium was the most common cause of renal failure followed by phosphide aluminum, organophosphates, and multiple drug poisoning. To sum up, it is important to treat these patients with special attention due to the higher risk of renal failure. Incidence of renal failure in phosphide poisoning had the second place which may be due to its high mortality before taking any laboratory tests and the rapid and fatal course of it. It should be considered that the other contributing factors like diabetes, hypertension, and chronic renal failure can also affect renal function (17-19). If the patient is admitted as soon as possible the prevalence of renal failure and rhabdomyolysis will decrease. In this study, briefly the prevalence of renal failure in 1500 poisoned patients was 8.9% with the higher incidence about the opium, aluminum phosphide, multiple medications and organophosphate. Incidence of renal failure increased with age, in patients with rhabdomyolysis, those admitted to the hospital later, and when appropriate medical treatment was not administered. It is essential to pay attention to a poisoned patient with these risk factors and find a proper solution to reduce the incidence of renal failure.
